# Evaluation of the efficacy of a novel lumbar exoskeleton with multiple interventions for patients with lumbar disc herniation: a multicenter randomized controlled trial of non-inferiority

**DOI:** 10.3389/fbioe.2024.1520610

**Published:** 2025-01-27

**Authors:** Xiaonan Huang, Lele Huang, Lei Shi, Lifan Xu, Chengbing Cao, Heng Wu, Min Cao, Can Lv, Ping Shi, Guohui Zhang, Fanfu Fang

**Affiliations:** ^1^ School of Health Sciences and Engineering, University of Shanghai for Science and Technology, Shanghai, China; ^2^ Department of Rehabilitation Medicine, Changhai Hospital, Naval Medical University, Shanghai, China; ^3^ School of Sports and Health, Shanghai University of Sport, Shanghai, China; ^4^ Yueyang Hospital of Integrated Traditional Chinese and Western Medicine Affiliated to Shanghai University of Traditional Chinese Medicine, Shanghai, China

**Keywords:** lumbar disc herniation, lumbar spine exoskeleton, lumbar traction, range of motion, conservative treatment

## Abstract

**Background:**

Lumbar disc herniation (LDH) occurs when the nucleus pulposus or annulus fibrosus protrudes into the intervertebral space, potentially compressing nerve roots and causing symptoms such as sciatica, restricted mobility, and lower extremity weakness. The development of portable lumbar exoskeleton devices offers a new approach, combining traction, range of motion (ROM) exercises, and resistance training in a single system, potentially reducing treatment complexity and enhancing LDH patient outcomes.

**Objective:**

This study aims to evaluate the efficacy and safety of a novel lumbar exoskeleton device compared to traditional traction methods combined with rehabilitation therapy for patients with LDH.

**Methods:**

A multicenter, non-inferiority randomized controlled trial was conducted with 118 participants diagnosed with LDH. Participants were randomly assigned to the Exoskeleton Group or the Traction Group. The Exoskeleton Group used the novel device for traction, ROM, and resistance training, while the Traction Group underwent traditional traction and rehabilitation therapy. Outcomes included efficacy rate after 10 treatments, Visual Analogue Scale (VAS), Oswestry Disability Index (ODI), and lumbar ROM—were assessed at 3, 6, and 10 treatments.

**Results:**

A total of 118 eligible participants were recruited. After 10 treatments, both groups showed significant improvements in VAS scores, ODI, and lumbar ROM compared to baseline (*P* < 0.001). However, there was no significant difference in the overall efficacy rate between the two groups (*P* = 0.748).

**Conclusion:**

The novel lumbar exoskeleton device demonstrates comparable efficacy and safety to traditional traction therapy combined with rehabilitation, offering a promising alternative for the conservative treatment of LDH.

## 1 Introduction

Lumbar disc herniation (LDH) refers to the protrusion of disc material—either the nucleus pulposus or annulus fibrosus—into the intervertebral space, which may or may not manifest with clinical symptoms on Magnetic Resonance Imaging (MRI). The prevalence of symptomatic LDH is estimated to range from 1% to 3%, with a higher incidence observed in individuals aged 30–60 years and a male-to-female ratio of 2:1 (Jordan et al., 2011). Several risk factors, such as smoking (OR 1.7, 95% CI 1.0–2.5), heavy lifting, and prolonged sitting, contribute to the development of LDH (Kelsey et al., 1984). The compression of nerve roots by the protruding disc material may result in symptoms such as sciatica, restricted mobility, and lower extremity weakness (Chou and Huffman, 2007). The WFNS Spine Committee currently recommends conservative treatment as the first-line approach for LDH, and a combination of physical therapy and exercise therapy can improve symptoms in most LDH patients (Ma et al., 2021; Yaman et al., 2024).

Studies report that 76.6% of patients with LDH have undergone lumbar traction therapy (Madson and Hollman, 2015). MRI findings confirm that even a single session of lumbar traction can modify intervertebral disc morphology, reduce the volume of herniated nucleus pulposus, separate the disc from adjacent nerve roots, and increase the facet joint space (Chung et al., 2015; Liu Z. Z. et al., 2021). Furthermore, lumbar length and disc realignment ratios are significantly improved during traction (Chung et al., 2015). However, the evidence for lumbar traction’s efficacy in alleviating back pain remains inconsistent. Some studies indicate no significant relief from lumbar traction and recommend against prioritizing it as a primary treatment option (Beurskens et al., 1995; Thackeray et al., 2016). While moderate-quality evidence supports lumbar traction’s short-term symptomatic relief of back pain, long-term benefits are not well-substantiated (Hahne et al., 2010). Traditional traction devices are typically bulky and require administration in specialized healthcare facilities (Hahne et al., 2010). Given the transient effects of traction, patients often need frequent and prolonged treatments, increasing their time and travel burdens (Fidelis, 2022). Additionally, the exclusive use of traction may neglect core muscle training, potentially leading to trunk muscle atrophy (Cholewicki et al., 2009). Consequently, lumbar traction is frequently combined with other physical therapies and postural training, adding complexity and skill requirements to the treatment regimen (Cholewicki et al., 2009). Finally, three clinical studies have reported adverse events associated with lumbar traction, including anxiety in patients undergoing inversion traction, with a subset experiencing lower limb weakness or fainting during treatment sessions (Liu and Zhang, 2000; Ozturk et al., 2006; Güevenol et al., 2009).

To address these limitations, we have developed an innovative lumbar exoskeleton device. This exoskeleton features a coupled rigid-flexible parallel structure with interactive capabilities (Wang Z. et al., 2022). In addition to conventional lumbar traction, the device supports six-degree-of-freedom mobility training and resistance exercises for the lumbar spine (Wang Q. et al., 2022). It also offers lumbar support to offload the injured core muscles, reducing the risk of secondary injury (Du et al., 2023). The exoskeleton is equipped with multiple adjustable prescription modes, allowing physicians or therapists to customize treatment plans. Once set, patients can perform treatments independently in community or home settings, minimizing the complexity of care and reducing time and travel costs. This study aims to compare the efficacy and safety of the novel lumbar exoskeleton versus traditional traction methods combined with conventional rehabilitation therapy, further evaluating its potential to alleviate the symptoms of lumbar disc herniation.

## 2 Materials and methods

### 2.1 Study design

This study is a multicenter non-inferiority randomized controlled trial. Participants were recruited from the department of rehabilitation medicine in two hospitals. Eligible participants were patients diagnosed with LDH accompanied by clinical symptoms. The trial is registered with the Clinical Trial Registry.

### 2.2 Eligibility criteria

Participants met the diagnostic criteria for LDH as defined by the Guidelines for the Diagnosis and Treatment of LDH (Association and Association, 2020). Inclusion criteria were as follows: meeting the diagnostic criteria for LDH, primarily experiencing low back or lumbosacral pain with a Visual Analogue Scale (VAS) pain score between 4 and 8; aged between 20 and 70 years; suitable for conservative treatment; and not having received medication, manual therapy, or traction treatment within 4 weeks prior to enrollment. Exclusion criteria included: patients with tumors, tuberculosis, spinal osteomyelitis, spinal fractures, cauda equina syndrome, or ankylosing spondylitis; those with non-discogenic causes of disease (e.g., spinal stenosis, inflammation, tumors), nonspecific low back pain, or acute low back pain; patients with severe cardiovascular, cerebrovascular, liver, or kidney diseases that severely threaten life, as well as those with mental illnesses; pregnant or breastfeeding women; patients with cognitive or communication impairments; and patients with osteoporosis. Shedding and Elimination criteria: failure to adhere to the rehabilitation training protocol; poor compliance during the trial, affecting the evaluation of efficacy and safety; occurrence of serious adverse events making continued participation inappropriate; voluntary withdrawal; loss to follow-up or death; and incomplete data affecting the evaluation of efficacy and safety.

### 2.3 Procedures

This study is divided into two groups: the Exoskeleton Group and the Traction Group. The exoskeleton group utilizes a novel lumbar exoskeleton device, while the traction group uses a traditional traction device (ASTAR TM300, Ito Co., Ltd.) combined with rehabilitation therapy.

**FIGURE 1:**
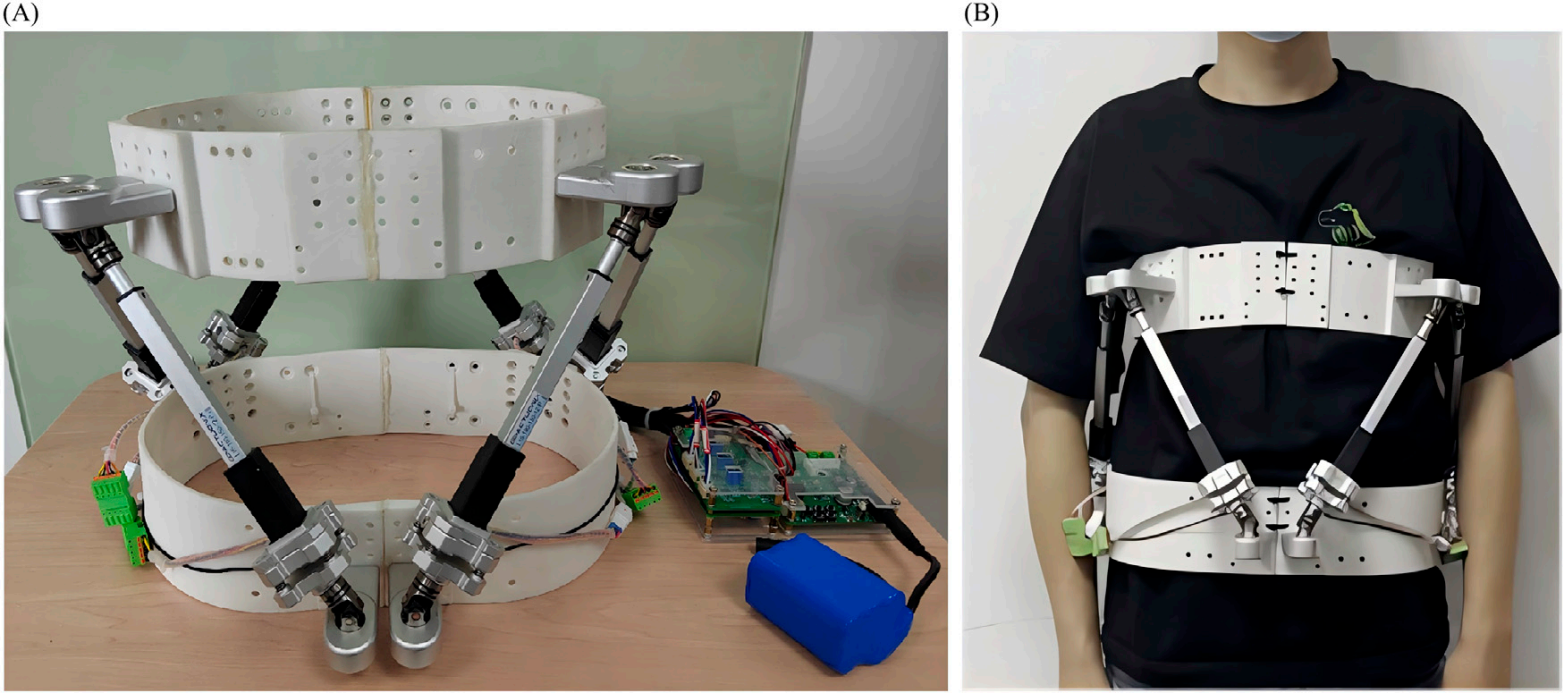
**(A)** A wearable lumbar exoskeleton traction device and **(B)** Subject undergoing rehabilitation training with a wearable lumbar exoskeleton traction device.

Exoskeleton Group: 1) Participants perform lumbar traction in a sitting position, wearing the exoskeleton securely, with the traction bands fastened to the lumbar region (Figure 1A). The traction intensity is set to a level where participants feel a noticeable stretch. Each session lasts 20 min 2) ROM training is conducted in a standing position under the guidance of the exoskeleton (Figure 1B). Participants perform six-directional ROM exercises targeting lumbar flexion, extension, lateral flexion to both sides, and rotation to both sides. Each direction is repeated 20 times, with the goal of achieving the maximum safe ROM. 3) Resistance training is performed with participants wearing the exoskeleton, executing exercises in six directions. Each direction involves three repetitions, repeated for a total of four sets. The resistance intensity is set at 2–5 kg for flexion and lateral flexion, 2–4 kg for rotation, and 5–10 kg for extension. A professional physical therapist determines the resistance levels to ensure the exercises are both safe and effective. The specific working principle and kinematic analysis of the equipment are detailed in the literature (Wang W. et al., 2022; Wang Z. et al., 2022; Du et al., 2023).

Traction Group: 1) Traction therapy is performed in a supine position, with the lumbar region secured using traction belts. The initial traction is set at 25% of the participant’s body weight and is gradually increased with each session, not exceeding 50% of their body weight. Each session lasts 20 min (Cheng et al., 2020). 2) Manual therapy involves a combination of spinal segment pressure, joint mobilization, and apex board techniques. Spinal segment pressure is applied by the therapist using their thumb or elbow on the affected disc segments. Joint mobilization is performed in a side-lying position, where the therapist places one hand on the lumbar segment and the other on the participant’s upper ankle, applying gentle force to relieve adhesions. The apex board technique is used to release tension in the lumbar and sacral regions. These interventions aim to improve joint mobility, release adhesions, and reposition the herniated disc to alleviate nerve root compression. Each session lasts 15 min (Qi et al., 2023). 3) Resistance training is performed under therapist supervision: For flexion, participants lie supine with knees bent at 90° and feet flat on the floor, crossing their arms over their chest and flexing forward while the therapist provides resistance at the shoulders or chest. For extension, participants lie prone with their hands behind their head, and the therapist applies resistance at the scapular area as they lift their upper body. Lateral flexion is performed in a standing position with arms relaxed at the sides, with the therapist providing resistance at the shoulder while participants bend to one side. Rotation is conducted in a seated position with feet flat on the ground, where participants rotate their trunk while the therapist applies counter-resistance at the shoulders or upper body. Each direction is performed for 12 repetitions per set, with two sets per session.

The above two groups of treatment were carried out every 2 days, for a total of 10 times.

This structured approach ensures that both the exoskeleton and traction groups receive consistent, comparable interventions. The exoskeleton group emphasizes active participation through ROM and resistance training with external support, aiming to improve function, flexibility, and strength. In contrast, the traction group focuses on passive decompression combined with manual therapy to target pain relief and joint mobility. The resistance training in both groups promotes the development of trunk muscle strength, which is essential for reducing the risk of future injuries and preventing recurrence of lumbar disc herniation.

### 2.4 Sample size and randomization

According to similar studies, the efficacy rate of combined manual therapy and traction for treating LDH was 94.64% in the trial group and 88.5% in the control group (Liu K. et al., 2021; Wang and Wang, 2021). Based on treatment methods and clinical experience, we set the efficacy rate of the control group at 94% and the trial group at 88%, considering a dropout rate of 10%. Using the non-inferiority trial sample size estimation method with α = 0.025, β = 0.2, and δ (non-inferiority margin) = 0.2, the required sample size was calculated to be 118 participants, with equal numbers in the exoskeleton and traction groups. Yueyang Hospital recruited 64 participants, while the First Affiliated Hospital of Naval Medical University recruited 54 participants.

All participants were randomly assigned using a random number table generated by SPSS 27. Random assignment cards were created, sealed in opaque envelopes, with the envelope numbers corresponding to the card numbers. Participants opened the envelope with the corresponding number in the order of their visits and were randomly assigned to the respective treatment groups as specified by the card inside the envelope. The random number table assigned participants to either the exoskeleton and traction groups in a 1:1 ratio.

### 2.5 Outcomes

#### 2.5.1 Primary outcome

The efficacy rate after 10 treatments: VAS score improvement rate = [(VAS score before treatment - VAS score after treatment) ÷ VAS score before treatment] × 100%. Markedly Effective: Symptoms significantly relieved, local pain significantly improved, VAS score improvement rate >60%; Effective: Symptoms somewhat improved, local pain somewhat relieved, VAS score improvement rate 30%–60%; Ineffective: No improvement in symptoms or signs, VAS score improvement rate<30%.

#### 2.5.2 Secondary outcomes

VAS: Pain levels assessed before treatment and after 3, 6, and 10 treatments.

ODI: Functional disability assessed using the ODI questionnaire before treatment and after 3, 6, and 10 treatments.

Lumbar ROM: Measured before treatment and after 3, 6, and 10 treatments, assessing the range of flexion, extension and lateral flexion of the lumbar spine.

## 3 Statistical analysis

Normality tests were conducted for all baseline data. For data conforming to a normal distribution, results were expressed as mean ± standard deviation (Mean ± SD); for non-normally distributed data, results were presented as median and interquartile range (Median and IQR). Baseline characteristics of the exoskeleton and traction groups were compared using independent samples t-test and chi-square test. For normally distributed continuous variables, independent samples t-test was used; for non-normally distributed continuous variables, the Mann-Whitney U test was employed; for categorical variables, chi-square test was utilized.

The primary outcome measure was the efficacy rate after 10 treatments, assessed by the VAS score improvement rate. Chi-square tests were used to compare the efficacy rates between the two groups. Secondary outcome measures included VAS scores, ODI, and lumbar ROM, evaluated before treatment and after 3, 6, and 10 treatments. Repeated Measures ANOVA was employed to assess the effects of time, group, and the interaction between time and group for these repeated measures data. To control for the error rate associated with multiple comparisons, Bonferroni correction was applied for *post hoc* tests. All statistical analyses were performed using SPSS 27, with the significance level set at 0.05, employing two-sided tests.

## 4 Result

**FIGURE 2:**
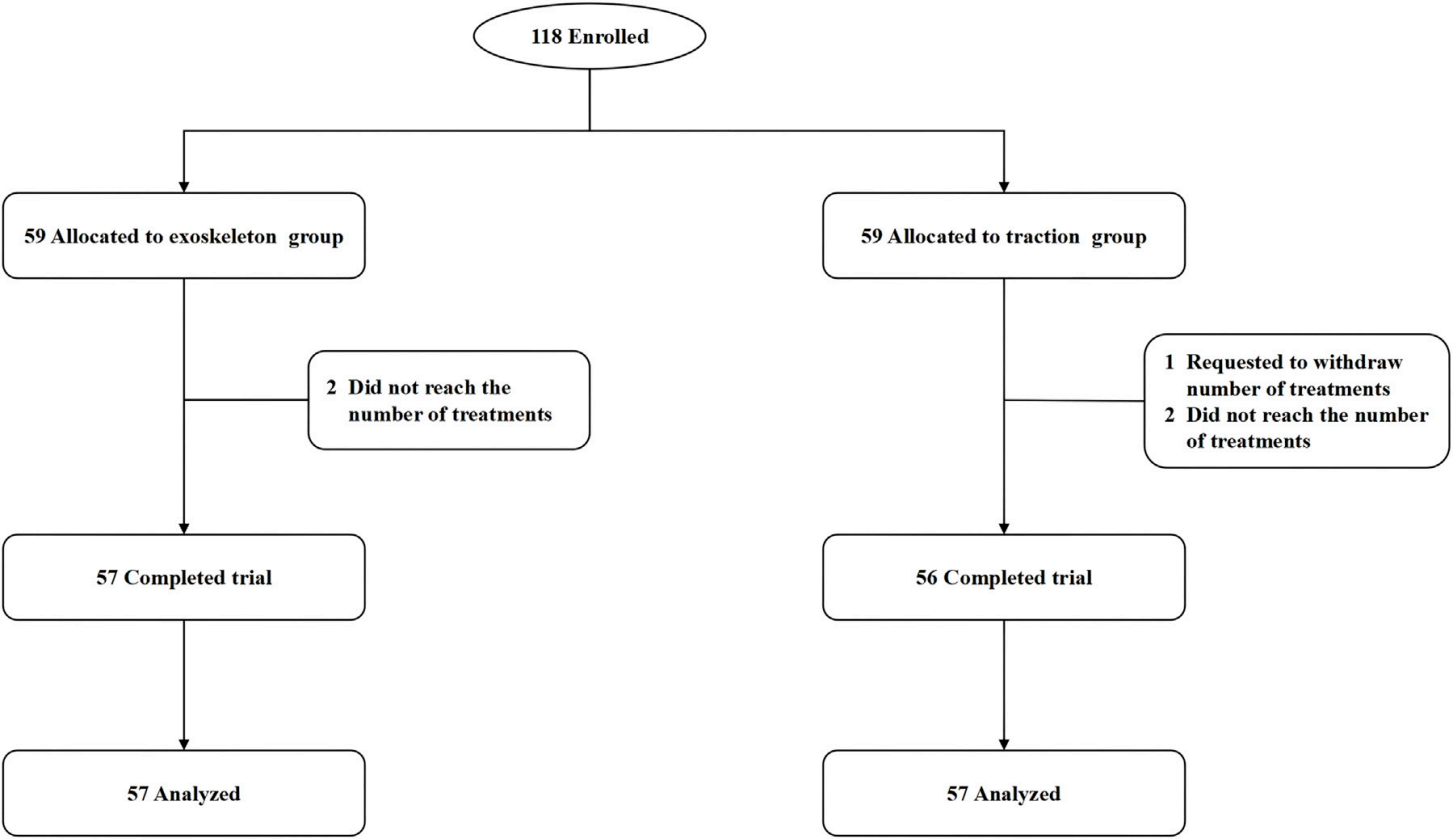
Patient enrollment flowchart.

During the period from December 2022 to January 2024, this study recruited 118 eligible subjects, with two dropouts in the exoskeleton group and three in the traction group. A total of 113 subjects completed the trial and were included in the analysis, as illustrated in Figure 2. Baseline characteristics of the two groups, including age, gender, height, weight, duration of illness, heart rate, history of low back pain, and nature of work, showed no statistically significant differences (Table 1).

**TABLE 1:** Baseline characteristics.

Variable	Participants		*P* value
	Exoskeleton Group (n = 60)	Traction Group (n = 60)	
Age	43.12 ± 13.35	40.7 ± 15.03	0.366
height	168 (161.5,175)	170.5 (165.25,175)	0.115
weight	65 (60.75,70.25)	65.25 (60.5,73)	0.874
BMI	22.94 (21.6,24.73)	22.76 (21.12,24.44)	0.425
Course of disease	10 (6,13)	8 (6.25,11)	0.119
Heart	72.79 ± 6.12	74.55 ± 5.22	0.102
Sex			0.453
Male	24	28	
Female	33	28	
History of chronic low back pain			0.288
Yes	45	39	
No	12	17	
Nature of work			0.449
Sedentary	30	25	
Regular	21	24	
Prolonged standing	3	6	
Physical activity	3	1	

BMI, indicates Body Mass Index.

The primary outcome measure, namely the therapeutic effect after 10 courses of treatment, showed no significant difference between the exoskeleton and traction groups (Table 2). In the exoskeleton group, 50 subjects (83.3%) showed marked improvement, 6 subjects (10%) showed improvement, and 1 subject (1.7%) showed no improvement. In the traction group, 47 subjects (78.3%) showed significant improvement, 8 subjects (13.3%) showed improvement, and 1 subject (1.7%) showed no improvement (*P* = 0.788). The efficacy rate in the exoskeleton group was 98.25%, while in the traction group it was 98.21% (*P* = 0.748).

**TABLE 2:** Effectiveness after ten treatments.

Variable	Exoskeleton Group (n = 60)	Traction Group (n = 60)	*P* value
Therapeutic effect			0.788
Efficacious	50	47	
Effective	6	8	
Ineffective	1	1	
Effectiveness rate	98.25%	98.21%	0.748

**FIGURE 3:**
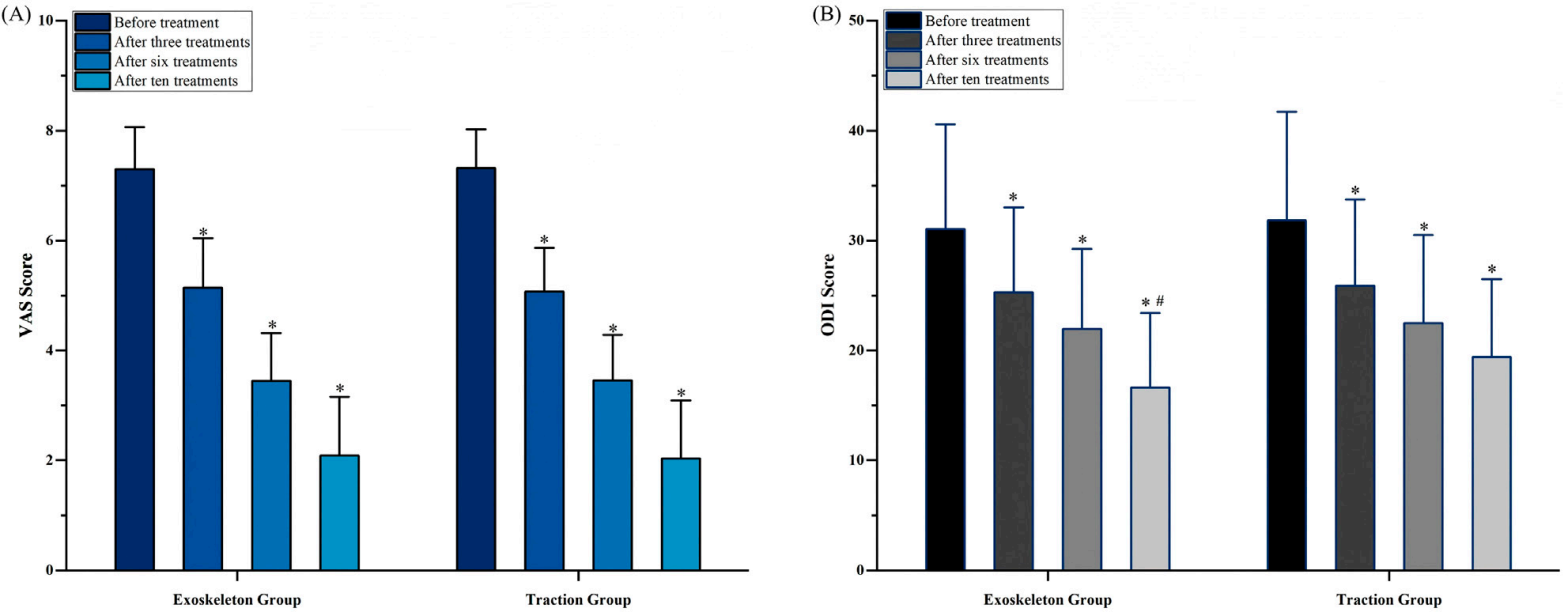
**(A)** VAS score and **(B)** ODI score. * compared to the previous measurement, P < 0.05. # compared to the Traction Group, P < 0.05. VAS indicates Visual Analogue Scale; ODI indicates Oswestry Disability Index.

Secondary outcomes included VAS scores, ODI, and lumbar spine mobility (flexion, extension, right lateral flexion, and left lateral flexion) after 3, 6, and 10 treatments. These results are presented in Table 3. Regarding VAS scores, both groups demonstrated significant improvement at each time point compared to baseline (*P* < 0.01, Figure 3A). However, there was no significant difference between the groups at each time point (*P* = 0.848). For ODI, both groups showed significant improvement at each time point compared to baseline (*P* < 0.01). At the measurement after 10 treatments, the ODI score was significantly lower in the exoskeleton group (16.64 ± 6.75) compared to the traction group (9.40 ± 7.10) (*P* = 0.036, Figure 3B). However, overall, there was no significant difference between the groups (*P* = 0.416). Regarding lumbar flexion, both groups showed significant improvement at each time point compared to baseline (*P* < 0.01), with no significant difference between the groups (*P* = 0.633). For lumbar extension, both groups demonstrated significant improvement at each time point compared to baseline (*P* < 0.01), with no significant difference between the groups (*P* = 0.375). For right lateral flexion, there was no significant difference in angle after 3 treatments in the traction group compared to before treatment, but improvement was observed at other time points (*P* < 0.01), with no significant difference between the groups (*P* = 0.374). For left lateral flexion, there was no significant difference in angle after the sixth treatment compared to after the third treatment in both groups, with improvement observed after 3 and 10 treatments compared to baseline (*P* < 0.01), and no significant difference between the groups (*P* = 0.512).

**TABLE 3:** Secondary outcome measures.

Outcome	Group	Before treatment	After three treatments	After six treatments	After ten treatments	*P* value^b^
VAS	Exoskeleton Group	7.32 ± 0.71	5.07 ± 0.80*	3.46 ± 0.83*	2.04 ± 1.05*	0.848
	Traction Group	7.30 ± 0.76	5.14 ± 0.90*	3.45 ± 0.87*	2.09 ± 1.07*	
ODI	Exoskeleton Group	31.82 ± 9.90	25.89 ± 7.85*	22.49 ± 8.02*	19.40 ± 7.10*	0.416
	Traction Group	31.04 ± 9.54	25.29 ± 7.75*	21.96 ± 7.26*	16.64 ± 6.75*^a^	
Forward flexion	Exoskeleton Group	23.93 ± 6.04	31.98 ± 4.32*	37.56 ± 4.85*	49.91 ± 3.86*	0.633
	Traction Group	24.39 ± 6.43	32.43 ± 4.13*	36.80 ± 4.53*	50.68 ± 3.98*	
Back extension	Exoskeleton Group	17.54 ± 4.64	21.84 ± 2.56*	28.14 ± 3.88*	32.28 ± 2.28*	0.375
	Traction Group	17.55 ± 4.63	21.86 ± 2.78*	27.02 ± 4.17*	32.39 ± 2.86*	
Right flexion	Exoskeleton Group	17.53 ± 5.28	25.05 ± 5.47*	30.42 ± 10.25*	39.75 ± 9.76*	0.374
	Traction Group	18.52 ± 3.95	19.30 ± 4.82	31.04 ± 6.20*	41.13 ± 9.81*	
Left flexion	Exoskeleton Group	15.63 ± 4.65	23.04 ± 4.28*	24.88 ± 4.38	40.47 ± 11.57*	0.512
	Traction Group	15.04 ± 3.61	23.36 ± 4.60*	23.86 ± 2.92	39.93 ± 12.04*	

*P < 0.05: Compared to the previous measurement, *P* < 0.05.

^a^ Compared to the Exoskeleton Group, *P* < 0.05.

^b^ Inter-group *P* value; VAS, indicates Visual Analogue Scale; ODI, indicates Oswestry Disability Index.

## 5 Discussion

This study is a multicenter non-inferiority randomized controlled trial aimed at evaluating the efficacy of a novel portable lumbar exoskeleton device for patients with LDH. The results indicate that at the end of treatment, there were no significant differences in treatment outcomes between the exoskeleton group and the traction group, suggesting that the multifunctional treatment provided by the novel lumbar exoskeleton is comparable in efficacy to traditional traction devices combined with rehabilitation therapy. After three, six, and ten sessions, there was a noticeable improvement in VAS, ODI, and lumbar ROM, indicating that both the lumbar exoskeleton device and traditional combined treatment can significantly alleviate symptoms and improve functional status in LDH patients. The ODI score for the Exoskeleton Group was significantly higher than that the exoskeleton’s combined approach—integrating lumbar traction, range-of-motion training, and resistance exercises—could lead to more comprehensive recovery, emphasizing both symptom relief and the enhancement of core muscle strength. Although the study period was relatively short, the active participation encouraged by the exoskeleton device likely promotes better muscle engagement and spinal stability, which could be pivotal for long-term functional benefits.

Traction therapy alleviates nerve root compression by relaxing the joint capsules, ligaments, and muscles surrounding the lumbar region, thereby expanding the intervertebral space and relocating the herniated material (Liu and Zheng, 2021). Additionally, traction can stimulate the release of endogenous analgesic substances (Liu and Zheng, 2021). For patients experiencing acute sciatica due to LDH, significant pain relief and improved daily activities were reported after 2 weeks of lumbar traction therapy (Isner-Horobeti et al., 2016). Meta-analyses indicate that lumbar traction can significantly improve pain and ODI scores in LDH patients, although it does not have a notable impact on spinal ROM (Vanti et al., 2021). The efficacy of lumbar traction is rapid, with immediate improvements in pain and function after a single session, but its effects are limited in duration, necessitating frequent treatments (Tanabe et al., 2021). At the same time, resistance training and mobility training play a significant role in the treatment of LDH. Research indicates that resistance training effectively enhances the strength and endurance of trunk and core muscles, improves body stability, and alleviates pain associated with LDH (Hlaing et al., 2021). Furthermore, by improving muscle function and postural control, resistance training reduces discomfort in the lumbar spine and enhances patients’ ability to perform daily activities (Kernc et al., 2018). Concurrently, mobility training focuses on increasing spinal flexibility and ROM, decreasing muscle stiffness, and optimizing neuromuscular coordination (Owen et al., 2020). One study highlighted that lumbar extension exercises not only enhance core strength but also play a positive role in the rehabilitation of patients with LDH (Zhang et al., 2019). Therefore, combining lumbar traction with rehabilitation therapy can significantly reduce lower back pain, enhance lumbar mobility, strengthen core muscles, and improve long-term outcomes (Wang and Wang, 2021; Tian, 2024).

Current mainstream lumbar exoskeleton devices primarily aim to improve human biomechanics, assisting in lifting heavy objects (Zhang and Huang, 2018) or providing passive support to the spine to reduce the effort exerted by injured trunk muscles (Koopman et al., 2020). While these devices help alleviate physical loads on the lower back and enhance overall stability during vigorous activities, their application in therapeutic interventions has not been widely implemented. Previous teams have developed a similar exoskeleton device designed to alleviate back pain through the provision of extensor torque and lumbar traction, finding that its use reduced electromyography (EMG) activation of the erector spinae muscles to minimize muscle fatigue (Moon et al., 2022). This study is the first to utilize a portable lumbar exoskeleton device in a periodic treatment regimen to improve pain and functional levels in LDH patients. The portable lumbar exoskeleton device features a 4-SPS/SP parallel mechanism design and a modular control system. It not only provides separation traction for the lumbar spine, expanding the intervertebral space to relieve nerve root compression, but also facilitates six degrees of freedom for assisted ROM training and resistance training through multi-directional levers, thereby enhancing lumbar ROM and trunk muscle strength to reduce spinal stress (Du et al., 2023). The integration of these functions allows LDH patients to perform traction, mobility training, and resistance training using a single exoskeleton device, reducing the complexity and expertise of treatment and increasing portability compared to traditional combined therapies. We previously found that core muscle electrical signals were significantly reduced when using the new exoskeleton device during activities, indicating its potential to help LDH patients alleviate lumbar load during pain (Wang Z. et al., 2022).

Lumbar ROM is closely correlated with pain levels, as patients with low back pain exhibit reduced lumbar flexion and extension angles (Coyle et al., 2017). This limitation may restrict patients’ daily activities, adversely affecting their quality of life and functional status. During episodes of back pain, patients often experience muscle tension, joint stiffness, and fear of movement, which can further exacerbate limitations in lumbar mobility, creating a vicious cycle (Knezevic et al., 2021). Therefore, alleviating pain is crucial for improving lumbar mobility. Consequently, we incorporated assisted mobility training into the exoskeleton device, and the results confirmed that effective pain relief can, to some extent, enhance lumbar mobility.

Adverse reactions to lumbar traction have been reported, such as muscle and ligament tears, vertebral fractures, and dizziness (Wei et al., 2003). In this study, no serious adverse reactions occurred in either group of participants. Some participants reported mild adverse reactions: specifically, two in the traction group reported muscle soreness, three reported lower limb weakness and dizziness, and one in the exoskeleton group reported muscle soreness. Muscle soreness is a result of the periodic resistance training leading to muscle maladaptation, as well as the stretching and friction of the muscles during the exoskeleton traction. However, the low incidence of adverse reactions also indicates a high level of device safety, suggesting that it can be used for treatment in home or community settings.

## 6 Limitations

Firstly, this study did not include follow-up assessments of participants, limiting the ability to demonstrate the long-term efficacy of the portable lumbar traction device. Secondly, as a first-generation product, the specific traction force depends on solely on participants’ subjective perception of traction intensity. Lastly, while the portable lumbar exoskeleton device offers resistance training, this study did not evaluate or compare abdominal muscle engagement, which could provide further insight into its therapeutic impact. Future studies could incorporate MRI or CT imaging to directly observe intervertebral disc morphology, providing objective evidence to complement evidence. This addition would enhance the study’s robustness and support its findings.

## 7 Conclusion

The traction treatment, ROM exercises, and resistance training provided by the new portable lumbar exoskeleton traction device demonstrate comparable efficacy and safety to traditional combined treatment methods. At the conclusion of the treatment, LDH patients experienced a significant reduction in pain and an improvement in lumbar function.

## Data Availability

The original contributions presented in the study are included in the article/supplementary material, further inquiries can be directed to the corresponding authors.
